# High precision implicit function learning for forecasting supercapacitor state of health based on Gaussian process regression

**DOI:** 10.1038/s41598-021-91241-z

**Published:** 2021-06-08

**Authors:** Jiahao Ren, Junfei Cai, Jinjin Li

**Affiliations:** 1 National Key Laboratory of Science and Technology on Micro/Nano Fabrication, Shanghai Jiao Tong University, Shanghai, 200240 China; 2 Key Laboratory for Thin Film and Microfabrication of Ministry of Education, Department of Micro/Nano-Electronics, Shanghai Jiao Tong University, Shanghai, 200240 China

**Keywords:** Electrical and electronic engineering, Energy science and technology, Supercapacitors

## Abstract

State of health (SOH) prediction of supercapacitors aims to provide reliable lifetime control and avoid system failure. Gaussian process regression (GPR) has emerged for SOH prediction because of its capability of capturing nonlinear relationships between features, and tracking SOH attenuations effectively. However, traditional GPR methods based on explicit functions require multiple screenings of optimal mean and covariance functions, which results in data scarcity and increased time consumption. In this study, we propose a GPR-implicit function learning, which is a prior knowledge algorithm for calculating mean and covariance functions from a preliminary data set instead of screening. After introducing the implicit function, the average root mean square error (Average RMSE) is 0.0056 F and the average mean absolute percent error (Average MAPE) is 0.6%, where only the first 5% of the data are trained to predict the remaining 95% of the cycles, thereby decreasing the error by more than three times than previous studies. Furthermore, less cycles (i.e., 1%) are trained while still obtaining low prediction errors (i.e., Average RMSE is 0.0094 F and Average MAPE is 1.01%). This work highlights the strength of GPR-implicit function model for SOH prediction of energy storage devices with high precision and limited property data.

## Introduction

Supercapacitors (SCs) are widely utilized and deployed in various systems because of their high power density, long cycle life, and operability over a wider temperature range^1–4^. However, the performance of SCs is constrained by irreversible aging problems, which threatens their reliable operation and leads to unpredictable system failures^5–7^. State of health (SOH), an important indicator for managing health and avoiding system failures, has received much attention and its prediction methods are developed rapidly. The challenge of SOH prediction is that, for one thing, it is difficult to construct an accurate failure model to simulate a real complex degradation process with multiple physical and chemical reactions. For another thing, many important parameters are difficult to measure which require expensive test equipment. Therefore, the methods of predicting SOH usually focus on capturing the internal state from easily measured parameters^8^.

The SOH can be predicted mainly by two different methods: model-based and data-driven. Model-based method uses complex mathematical equations to simulate degradation mechanisms, necessitating a deeper understanding of electrochemical principles. Recently, various filtering models with model-based methods are applied in the previous research about the SOH prediction^9,10^. For example, Mejdoubi et al.^11^ proposed a particle filter (PF) model to estimate the SOH of SCs, in which the capacitance and resistance could be predicted accurately under various conditions by considering the aging temperature and voltage. Walker et al*.*^12^ compared the PF model with Kalman filter (KF) model for predicting the remaining useful life of a lithium-ion battery, and found the PF model to be superior to a KF model. Mohamed et al*.*^13^ proposed an enhanced mutated PF (EMPF) model for the SOH prediction of a lithium-ion battery and proved that the model can effectively capture dynamic system behaviors. These studies can effectively capture part of the dynamic behaviors of the system by establishing accurate mathematical models. However, such prediction methods have the following problems^14^: (1) The physical and chemical processes in energy storage devices are very complex. Therefore, establishing physical models of RUL and SOH for energy storage devices is quite difficult. (2) These methods cannot accurately simulate the real attenuation process, because the trajectory of the attenuation is affected by the surrounding environment and load conditions, which are constantly changing. (3) The model-based method is semi-empirical, so the corresponding results depend on the knowledge of the researchers and the quality of the experiment equipment.

Compared with the model-based methods, the data-driven methods focus on finding the interaction relationship between data and do not make any assumptions about the RUL degradation of the device. Data-driven methods avoid building accurate electrochemical models, and have attracted significant attention, gradually becoming the mainstream for SOH prediction^15^. Various machine learning methods have been proposed for predicting SOH, for example, linear or nonlinear regression models^16,17^, support vector machine method^18–21^, artificial neural network method^22,23^, long short term memory algorithm^24,25^, and so on. The goal of these models are the same: to find the mapping of input features for SOH prediction. However, the mapping from cycle of charging and discharging based on these conventional data-driven methods is over-simplistic due to the uncertainty of vectors in degradation process^26^. Compared to conventional data-driven methods, Gaussian process regression (GPR), a data-driven SOH prediction method, possesses hyper-parameter adaptive acquisition and can be easily implemented, does not involve parameters. Because of the non-parametric characteristic^27^ of GPR, the model can be calibrated naturally according to data requirements. Furthermore, GPR can not only give a specific output, but also provides confidence for the predicted results of the model, so as to make an informed decision. According to the research of Chen et al.^28^, GRP is very suitable for solving complex regression problems, including high-dimensional and nonlinear predictions. Therefore, GPR has attracted great attention in the prediction of SOH, which is a complex nonlinear regression problem. For example, Liu et al.^29^ applied linear and quadratic polynomial mean function with compound covariance function composed of squared exponential and periodic covariance function to predict the SOH of self-recharging lithium-ion batteries (the first 100 cycles for training, the rest 68 cycles for test). Richardson et al.^26^ used power function as a mean function and analyzed prediction results with different covariance functions, including squared exponential, periodic, and Matérn covariance functions (approximately the first 72 cycles for training, approximately the rest 108 cycles for test). Yang et al.^27^ selected four input features based on charging curves, analyzed these features based on gray relational analysis, and proposed a compound covariance function called double squared exponential for SOH prediction (the first 80 cycles for training, approximately the rest 90 cycles for test). These pioneering studies with satisfactory prediction accuracy are based on the explicit functions (i.e., having a specified equation) of GPR methods, and mainly focus on the selection of input features, mean function, and covariance function. In these studies, at least 40% of the data set were used as training data, which helps improve the accuracy of SOH prediction; however, finding the most suitable configuration is time-consuming. In addition, once the system changes, the previously selected input features, mean function, and covariance function are not necessarily applicable anymore, which means researchers must find different configurations for different systems. To solve the problem mentioned above and find GPR with higher efficiency and accuracy, numerous methods have been proposed to improve the performance of GPR (efficiency and accuracy) recent years. For example, Li et al*.* combined GPR with other regression methods and proposed a multi-time-scale framework for predicting the SOH and RUL of Lithium ion batteries^30^; Wang et al*.* used a modified kernel GPR algorithm to simulate the degradation process of batteries^31^; Hu et al*.* designed a dual GPR model for the SOH and RUL prognosis of battery packs^32^. These studies are all aimed at improving the accuracy and efficiency of the GPR methods in the prognosis of SOH and RUL, illustrating the necessity of modifying the GPR to obtain better performance.

**Figure 1:**
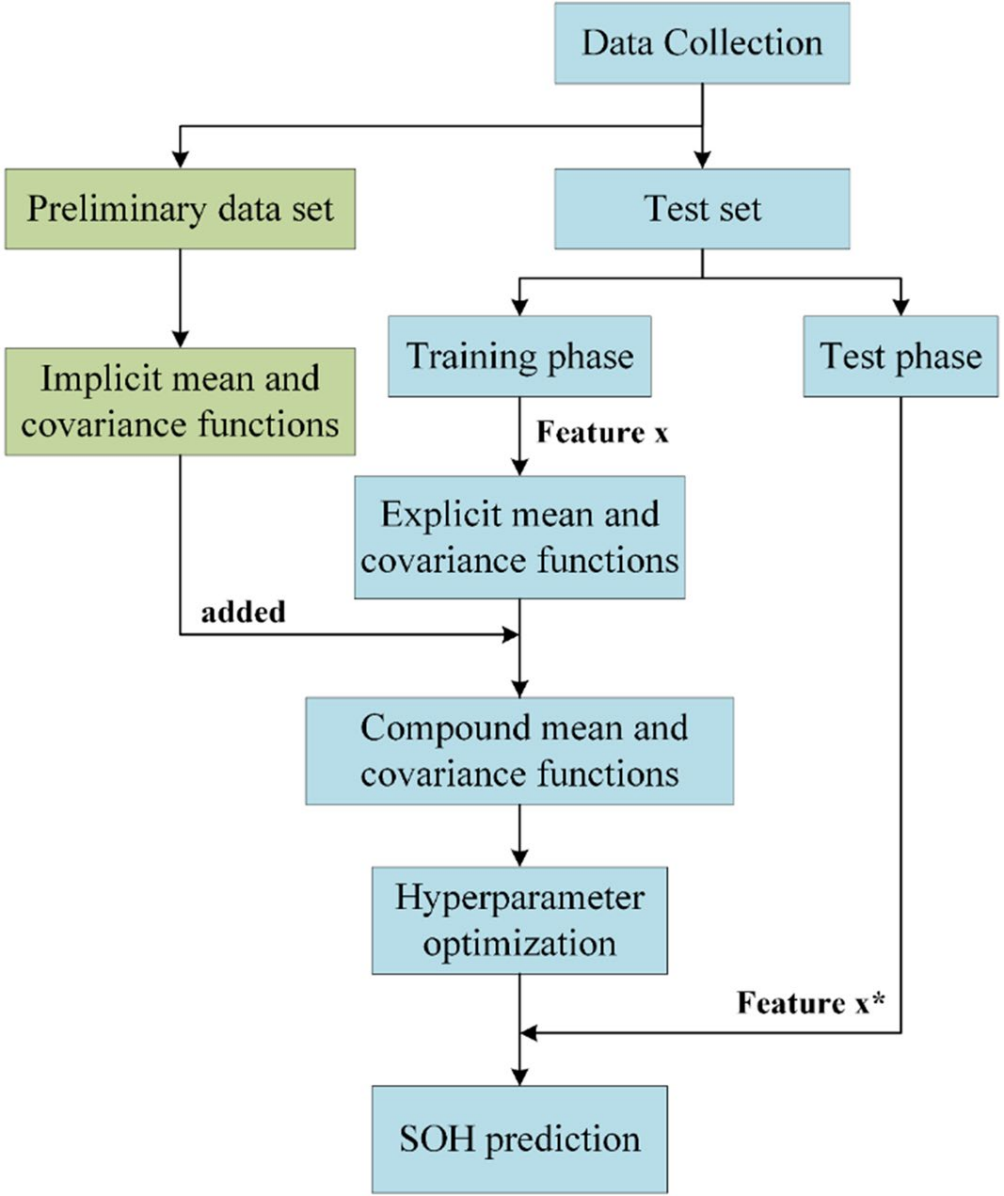
The workflow of SOH prediction. The blue parts correspond to the conventional methods of SOH prediction, where explicit functions are used in GPR. The green parts correspond to the GPR-implicit function model presented in this study, where the used implicit mean and covariance functions are derived from the preliminary data set.

In this study, we present a more general method based on the use of implicit mean and covariance functions of GPR methods. Specifically, this study presents the following procedure of SOH prediction: (1) a batch of SCs undergo cyclic charge and discharge tests (called preliminary data set), following which the capacitance after each cycle is collected to calculate the mean value of each cycle (regarded as the implicit mean function) and the covariance between a pair of cycles (regarded as the implicit covariance function)^33^; (2) the explicit and implicit mean and covariance functions are used to perform GPR predictions, respectively; and (3) the SOH predictions with reduced cycle data are discussed. The workflow of SOH prediction with a GPR-implicit function model is shown in Fig. 1, where the blue parts correspond to traditional SOH prediction with explicit functions and the green parts correspond to GPR-implicit function model proposed in this study. The compound mean and covariance functions indicate that the mean function and covariance function contain the explicit function and implicit function simultaneously. Since the test set contains 22 SCs and each SC will produce a RMSE and a MAPE, we use average root mean square error (average RMSE) and average mean absolute percent error (average MAPE) to reasonably evaluate models (see “Evaluation” section for details). In this study, the predicted Average RMSE is 0.0056 F and the Average MAPE is 0.6% after introducing the implicit function, where only the 5% of the cycles are used as the training data to predict the remaining 95% of the cycles. Compared with the previous studies based on explicit functions, the GPR-implicit function model decreases the prediction error by more than three times. We further use less cycles as the training data, that is, 1% of the cycles, to predict the remaining 99% of the cycles, which also produce reasonable prediction errors (i.e., Average RMSE of 0.0094F and Average MAPE of 1.01%). Although only the univariate model, that is, capacitance vs. cycle, is applied, the GPR-implicit function model’s high prediction accuracy and good robustness with less training data highlights the ability of implicit functions to model the real SOH of SCs. The method proposed in this study is applicable not only to SOH prediction of SCs, but also to other energy storage devices such as lithium-ion batteries.

## Data preprocessing

The research framework is shown in Fig. S1 of Supplementary Materials (SM). As shown in Fig. S1a, the supercapacitors undergo cycles of charging and discharging, which provides a preliminary data set for the subsequent predictions of the Gaussian process. Then, based on the preliminary data set, we predict the SOH of supercapacitors by different Gaussian regression processes (i.e., with different mean or covariance functions), and obtained their RMPE and MAPE as the judging criteria for performance prediction. This comparison shows the precision advantage of GPR with implicit functions. Figure S1b shows the comparison of accuracy when using different proportions of data as the training set, where the GPR with an implicit function has a higher accuracy than conventional GPR even if the proportion of the training set is reduced. More details can be found in “Results and discussion” section.

### Definition of SOH

SOH is an indicator for characterizing the degree of aging in SCs, providing users with basic information on health status, thereby ensuring a stable operation of energy storage devices (i.e., SCs or lithium-ion batteries). There are two widely-adopted definitions of SOH: one based on equivalent series resistance (ESR) increase, and the other based on capacitance decrease^34,35^. The SOH in this study refers to the definition of capacitance ratio, which is expressed in Eq. (1), where *SOH*_*i*_ represents the SOH value of the *i*-th cycle, *C*_*i*_ is the capacitance of the *i*-th cycle, and *C*_*R*_ is the rated capacitance of the SC. As the number of cycles increase, the SC ages because of an irreversible reaction during the charge and discharge processes, leading to a decrease in capacitance. In fact, the rated capacitance of the SC used in this study is 1C; therefore, *SOH*_*i*_ and *C*_*i*_ are equal.

1 $$ \begin{array}{*{20}c} {SOH_{i} = \frac{{C_{i} }}{{C_{R} }}.} \\ \end{array} $$

### Experimental data and analysis

In this work, the cycle data of 88 SCs are obtained, and each SC is cycled 10,000 times with a charging and discharging policy in a temperature-controlled environment (28 ℃). All SCs are discharged at a constant current of 20 mA. To conform to the actual charging process, we apply two different charging policies for SCs. The first is a 20 mA constant current charging policy, and the second is a stepped charging policy which refers to a stepped current of 15 mA from 1 to 1.85 V, 10 mA from 1.85 to 2.36 V, and 5 mA from 2.36 to 2.7 V.

**Figure 2:**
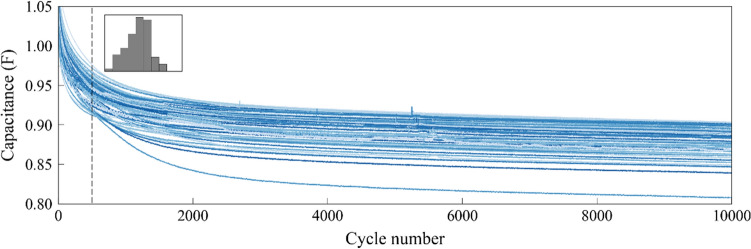
SOH trajectories of SCs as a function of cycle number, calculated from the preliminary data set. The inset shows the distribution of capacitance at 500th cycle which approximates the Gaussian distribution.

Preliminary data set refers to the data set of previous SCs that have been tested before a new SC cycle. For example, for a user who has tested 10 SCs, the cycle data for these 10 SCs can be used as the preliminary data set to predict the SOH of 11th SC. Figure 2 shows the SOH trajectories of preliminary data set of SCs used in this study, most of which intersect each other and follow a similar aging rate: from fast to slow. Such an aging behavior is different from the observed battery aging behavior^36,37^. To better understand the data from a statistical point of view, the inset of Fig. 2 presents the capacitance distribution of the 500th cycle, which is remarkably close to the Gaussian distribution. Such Gaussian distribution can also be observed in other cycles, which inspires us to use the GPR-implicit function model for predicting the SOH of SCs, according to the GPR assumption that each predicted output follows (i.e., capacitance) a Gaussian distribution, and any pair of outputs obey a joint Gaussian distribution. These concepts will be detailed in the subsequent sections.

**Figure 3:**
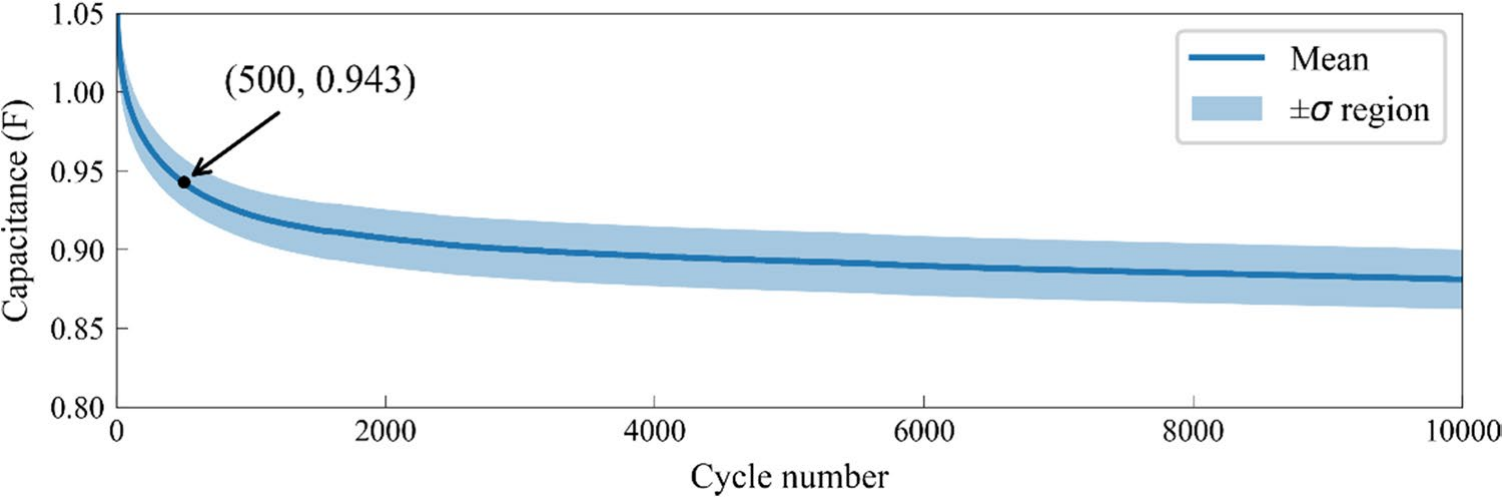
Mean capacitance as a function of cycle number, calculated from the preliminary data set. The black point indicates that the mean capacitance at 500th cycle is 0.943 F. The blue shaded area represents the positive and negative standard deviations of capacitance between mean-σ and mean + σ, where σ represents the capacitance dispersion degree of a given cycle, and is used to calculate the implicit covariance function in GPR.

Different from the explicit function used widely in GPR methods, the implicit function, coming from the preliminary data set, does not have a specific equation. To achieve a high-precision SOH prediction with appropriate implicit functions, we calculate the mean and standard deviation of capacitance for each cycle based on the attenuation curves of preliminary data set of 66 SCs. Figure 3 shows the mean capacitance (blue curve) and its positive and negative standard deviations (blue shaded area) as a function of cycle number, where the decay goes from fast to slow with increasing cycle number. The decay behavior in Fig. 3 is the same as in Fig. 2. The standard deviation in Fig. 3 quantifies the dispersion degree of capacitance of each cycle in Fig. 2, which can be used to calculate the implicit covariance function in GPR. Specifically, the average capacitance of the 500th cycle calculated from 66 SCs is 0.943 F, corresponding to a standard deviation of 0.015 F, which is shown as the black point in Fig. 3. Therefore, the implicit mean function and covariance function can be obtained from the preliminary data set for high-precision SOH predictions.

## Method

GPR is a nonparametric stochastic process based on probability^38^. Unlike most machine learning algorithms that give only a specific output, GPR gives the probability distribution of an output based on the Bayesian theory. The key idea behind Bayesian theory is that the posterior probability can be modified based on prior probability after observing an event. Such a result gives credibility to the prediction from a probabilistic perspective, and provides more adequate prediction information.

### GPR

The goal of GPR is to learn the mapping from input vector *x* to some Gaussian distribution *f*(*x*), given the observed input–output data points , where *n* is the total number of training data points, $x_{i}$ is the *i*-th n-dimensional input vector, and *y*_*i*_ is the *i*-th output. In this study, $x_{i}$ refers to the *i*-th cycle number, and *y*_*i*_ refers to the *i*-th capacitance.

To obtain the mapping, a joint distribution among these distributions should be considered. That is, the properties of multivariate distribution $f\left( {\mathbf{x}} \right)$ can be completely determined by a mean function *m*(**x**) and a covariance function $\kappa \left( {{\mathbf{x}}, {\mathbf{x^{\prime}}}} \right)$, which is denoted as2where *m*(**x**) and $\kappa \left( {{\mathbf{x}}, {\mathbf{x^{\prime}}}} \right)$ are denoted by

3 $$ \begin{array}{*{20}c} {\left\{ {\begin{array}{*{20}l} {m\left( {\mathbf{x}} \right) = E\left[ {f\left( {\mathbf{x}} \right)} \right]} \hfill \\ {\kappa \left( {{\mathbf{x}},{\mathbf{x^{\prime}}}} \right) = E\left[ {\left( {f\left( {\mathbf{x}} \right) - m\left( {\mathbf{x}} \right)} \right)\left( {f\left( {{\mathbf{x^{\prime}}}} \right) - m\left( {{\mathbf{x^{\prime}}}} \right)} \right)} \right]} \hfill \\ \end{array} } \right..} \\ \end{array} $$

In a basic GPR, the mean function is usually set to zero, and the covariance function is usually composed of a squared exponential (SE) covariance function and noise function, as shown in Eq. (4).

4 $$ \begin{array}{*{20}c} {\kappa \left( {{\mathbf{x}},{\mathbf{x^{\prime}}}} \right) = \kappa_{SE} \left( {{\mathbf{x}},{\mathbf{x^{\prime}}}} \right) + \kappa_{n} \left( {{\mathbf{x}},{\mathbf{x^{\prime}}}} \right).} \\ \end{array} $$

$\kappa_{SE} \left( {{\mathbf{x}},{\mathbf{x^{\prime}}}} \right)$ denotes the *n* × *m* matrix of the covariance evaluated at all pairs of training and test points, where **x** contains *n* training points and **x′ **contains *m* test points. In practice, the covariance between two points, *x*_*i*_ and *x*_*j*_ can be calculated by

5 $$ \begin{array}{*{20}c} {k_{ij} = \sigma_{f}^{2} \exp \left( { - \frac{{x_{i} - x_{j}^{2} }}{{2l^{2} }}} \right),} \\ \end{array} $$

where ${\upsigma }_{f}$ and *l* are hyper-parameters for controlling the vertical scale and length scale of the Gaussian function, respectively. Many types of covariance functions exist, and most have their own application scenarios. These covariance functions can also be combined with each other by affine transformations to produce a compound function for discovering more subtle data rules. In essence, a covariance function describes the correlation strength between the inputs of two points, and will finally affect the predicted probability distribution through a joint distribution. This means the choice of covariance function plays a crucial role in the final prediction result.

In a regular regression problem, the output $y$ of the model consists of two parts as follows:

6 $$ \begin{array}{*{20}c} {y = f\left( x \right) + \varepsilon ,} \\ \end{array} $$

where $f\left( x \right)$ represents the actual rule and  is invisible noise. In GPR, $f\left( x \right)$ is the Gaussian process we expect to obtain. Here, the observed *y* is also a Gaussian process restricted by Gaussian joint distribution. Therefore, when calculating the *n*-dimensional symmetric positive definite covariance matrix of training points $\kappa \left( {{\mathbf{x}},{\mathbf{x}}} \right)$, the noise function is expectedly considered, and denoted as

7 $$ \begin{array}{*{20}c} {\kappa_{n} \left( {{\mathbf{x}},{\mathbf{x}}} \right) = \sigma_{n}^{2} I,} \\ \end{array} $$

where ${\upsigma }_{n}$ is a hyper-parameter of noise and **I** is a *n*-dimensional identity matrix.

After the covariance function is determined, the corresponding hyper-parameters,$\Theta = \left[ {\sigma_{f}^{2} , l, \sigma_{n}^{2} } \right]$, are optimized by maximizing the log-likelihood function with training points, which is defined as

8 $$ L = \log p\left( {{\mathbf{y}}{|}{\mathbf{x}}, \Theta } \right){ } = - \frac{1}{2}\log \left( {\det \left( {\kappa \left( {{\mathbf{x}},{\mathbf{x}}} \right) + \sigma_{n}^{2} {\mathbf{I}}} \right)} \right) - \frac{1}{2}{\text{y}}^{T} \left[ {\kappa \left( {{\mathbf{x}},{\mathbf{x}}} \right) + \sigma_{n}^{2} {\mathbf{I}}} \right]^{ - 1} {\mathbf{y}} - \frac{n}{2}{\text{log}}2\pi . $$

Based on the above implementation, the training phase of GPR is completed. Then, the posterior distribution of test points is derived from the conditions of prior distributions, joint distributions, and training points through the Bayesian formula for performing the regression prediction. Specifically, for a given set of inputs ${ }{\mathbf{x}}^{*}$ of test points, we assume that their corresponding outputs ${\mathbf{f}}^{*}$ also follow a Gaussian distribution, and are limited to the joint Gaussian distribution with training points. This relationship can be described as

9 $$ \begin{array}{*{20}c} {\left( {\begin{array}{*{20}c} {\mathbf{y}} \\ {{\mathbf{f}}^{*} } \\ \end{array} } \right)\sim \left( {\begin{array}{*{20}c} {0, \left( {\begin{array}{*{20}c} {\kappa \left( {{\mathbf{x}},{\mathbf{x}}} \right) + \sigma_{n}^{2} {\mathbf{I}}} & {\kappa \left( {{\mathbf{x}},{\mathbf{x}}^{*} } \right)} \\ {\kappa \left( {{\mathbf{x}}^{*} ,{\mathbf{x}}} \right)} & {\kappa \left( {{\mathbf{x}}^{*} ,{\mathbf{x}}^{*} } \right)} \\ \end{array} } \right)} \\ \end{array} } \right).} \\ \end{array} $$

The posterior distribution is derived as10

This means that when ${\mathbf{x}},{\mathbf{y}},{\mathbf{x}}^{*}$ are known, ${\mathbf{f}}^{*}$ obeys a multivariate Gaussian distribution with mean vector $\overline{{{\mathbf{f}}^{*} }}$ and covariance matrix ${\text{cov}}\left( {{\mathbf{f}}^{*} } \right)$, where

11 $$ \begin{array}{*{20}c} {\overline{{{\mathbf{f}}^{*} }} = \kappa \left( {{\mathbf{x}}^{*} ,{\mathbf{x}}} \right)\left[ {\kappa \left( {{\mathbf{x}},{\mathbf{x}}} \right) + \sigma_{n}^{2} {\mathbf{I}}} \right]^{ - 1} {\mathbf{y}},} \\ \end{array} $$

12 $$ \begin{array}{*{20}c} {cov\left( {{\mathbf{f}}^{*} } \right) = \kappa \left( {{\mathbf{x}}^{*} ,{\mathbf{x}}^{\user2{*}} } \right) - \kappa \left( {{\mathbf{x}}^{*} ,{\mathbf{x}}} \right)\left[ {\kappa \left( {{\mathbf{x}},{\mathbf{x}}} \right) + \sigma_{n}^{2} {\mathbf{I}}} \right]^{ - 1} \kappa \left( {{\mathbf{x}},{\mathbf{x}}^{*} } \right),} \\ \end{array} $$

when a specific predicted value is required, the mean set, $\overline{{{\mathbf{f}}^{*} }}$, is used as the output of GPR model. Meanwhile, the matrix ${\text{cov}}\left( {{\mathbf{f}}^{*} } \right)$ is used for determining the confidence area by the variance of output extracted from the matrix diagonal.

The input vector *x*_*i*_ and output *f*(*x*_*i*_) in this article are both one-dimensional scalars, where *x*_*i*_ is a time series, that is, the number of cycles^26,29^, rather than physical features^14,27,39,40^, and the output is the corresponding discharge capacitance. However, when needed, a similar study for obtaining better results can be performed in the case of a high-dimensional input, although it requires some feature engineering.

### Improved GPR

In most cases, especially for an interpolation prediction problem (the input of the test SC is located between the inputs of the training SC), setting the mean function to 0 is acceptable because of the flexibility of GP to model the real mean^41^. However, when the test input is far from the training input, such as a prediction problem based on time series, the prediction ability of GPR will be limited, and the prediction performance will decline rapidly with the increase of the distance. In this case, most covariance functions are difficult to obtain effective information, so the final prediction is mainly determined by the mean function, which is set as a constant of 0. For example, in the case of the basic GPR mentioned above, when the distance between $$x_{i}$$ (training input) and $$x_{j} $$(test input) is relatively large, the covariance term $$k_{ij}$$ is almost zero, resulting in the output $$y_{j}$$ being zero.

If the mean function is adjusted so that it can capture some rules embedded in the model, the prediction performance can be improved. Most existing studies mainly focus on parametric function fitting, whose mean function is obtained by fitting the training set, for example, using a linear function, a quadratic function, or other degradation formulas. However, for energy storage devices such as batteries and SCs, a parametric function describing the degradation process is difficult to determine, and it also can hardly describe the entire degradation process accurately, since degradation is a complex nonlinear process. Here, the mean function is improved by prior knowledge from the preliminary data set, rather than by curve fitting. Specifically, for a given input *x*_*i*_, its mean function is

13 $$ \begin{array}{*{20}c} {{\text{m}}_{pre} {(}x_{i} {) = }\frac{1}{N}\mathop \sum \limits_{{k \in D_{p} }} y_{i}^{\left( k \right)} ,} \\ \end{array} $$

where $D_{p}$ is the preliminary data set, $N$ is the size of preliminary data set, and $y_{i}^{\left( k \right)}$ is the observed output at *i*-th point of *k*-th SC. The mean function remains unchanged after the preliminary data set determined.

By adjusting the mean function in this way, the posterior distribution can similarly be derived from the prior distribution. The prior distribution is denoted as

14 $$ \begin{array}{*{20}c} {\left( {\begin{array}{*{20}c} {\mathbf{y}} \\ {{\mathbf{f}}^{*} } \\ \end{array} } \right)\sim \left( {\left( {\begin{array}{*{20}c} {{\text{m}}_{pre} \left( {\mathbf{x}} \right)} \\ {{\text{m}}_{pre} \left( {{\mathbf{x}}^{\user2{*}} } \right)} \\ \end{array} } \right)\begin{array}{*{20}c} {, \left( {\begin{array}{*{20}c} {\kappa \left( {{\mathbf{x}},{\mathbf{x}}} \right) + \sigma_{n}^{2} {\mathbf{I}}} & {\kappa \left( {{\mathbf{x}},{\mathbf{x}}^{\user2{*}} } \right)} \\ {\kappa \left( {{\mathbf{x}}^{*} ,{\mathbf{x}}} \right)} & {\kappa \left( {{\mathbf{x}}^{*} ,{\mathbf{x}}^{\user2{*}} } \right)} \\ \end{array} } \right)} \\ \end{array} } \right).} \\ \end{array} $$

And the posterior distribution is15

This means that when ${\mathbf{x}},{\mathbf{y}},{\mathbf{x}}^{*}$ are known, ${\mathbf{f}}^{*}$ obeys a multivariate Gaussian distribution with mean vector $\overline{{{\mathbf{f}}^{*} }}$ and covariance matrix ${\text{cov}}\left( {{\mathbf{f}}^{*} } \right)$, where

16 $$ \begin{array}{*{20}c} {\overline{{{\text{f}}^{*} }} = {\text{m}}_{pre} \left( {{\mathbf{x}}^{*} } \right) + \kappa \left( {{\mathbf{x}}^{*} ,{\mathbf{x}}} \right)\left[ {\kappa \left( {{\mathbf{x}},{\mathbf{x}}} \right) + \sigma_{n}^{2} {\mathbf{I}}} \right]^{ - 1} \left( {{\mathbf{y}} - {\text{m}}_{pre} \left( {\mathbf{x}} \right)} \right),} \\ \end{array} $$

17 $$ \begin{array}{*{20}c} {cov\left( {{\mathbf{f}}^{*} } \right) = \kappa \left( {{\mathbf{x}}^{\user2{*}} ,{\mathbf{x}}^{*} } \right) - \kappa \left( {{\mathbf{x}}^{*} ,{\mathbf{x}}} \right)\left[ {\kappa \left( {{\mathbf{x}},{\mathbf{x}}} \right) + \sigma_{n}^{2} {\mathbf{I}}} \right]^{ - 1} \kappa \left( {{\mathbf{x}},{\mathbf{x}}^{*} } \right),} \\ \end{array} $$

where the ${\text{cov}}\left( {{\mathbf{f}}^{*} } \right)$ is the same as Eq. (12). More details can be found in Ref.^37^.

The prior knowledge includes not only the mean but also the covariance matrix. The traditional covariance matrix is calculated by an explicit covariance function, such as the SE covariance function mentioned above. However, it is also difficult to determine the covariance function, which requires more modeling ability than the mean function. This is because it plays a bigger role in GPR. Since the mean function is obtained from the preliminary data set, a similar procedure can be used for the covariance function. For any two inputs *x*_*i*_ and *x*_*j*_, the covariance term is defined as

18 $$ k_{ij} = {\text{ m(}}x_{i} x_{j} {)} - {\text{ m(}}x_{i} {\text{)m(}}x_{j} {)} = \frac{1}{N}\mathop \sum \limits_{{k \in D_{p} }} y_{i}^{\left( k \right)} y_{j}^{\left( k \right)} - \frac{1}{{N^{2} }}\mathop \sum \limits_{{k \in D_{p} }} y_{i}^{\left( k \right)} \mathop \sum \limits_{{k \in D_{p} }} y_{j}^{\left( k \right)} . $$

A covariance function composed of these terms is called $\kappa_{pre}$. At this point, the final covariance function can be extended as follows:

19 $$ \begin{array}{*{20}c} {\kappa \left( {{\mathbf{x}},{\mathbf{x^{\prime}}}} \right) = \kappa_{pre} \left( {{\mathbf{x}},{\mathbf{x^{\prime}}}} \right) + \kappa_{SE} \left( {{\mathbf{x}},{\mathbf{x^{\prime}}}} \right) + \kappa_{n} \left( {{\mathbf{x}},{\mathbf{x^{\prime}}}} \right).} \\ \end{array} $$

The acquisition process of the above-mentioned mean and covariance functions is shown in Fig. S2, which corresponds to the green part of Fig. 1.

### Evaluation

The GPR model is evaluated using RMSE, MAPE, and confidence intervals. RMSE and MAPE are used to quantitatively evaluate the predicted capacitance error, reflecting the model accuracy. Given a certain cycle *c* as the dividing point, cycle *c* and cycles before *c* are the training phase, and cycles after *c* are the test phase. RMSE and MAPE only apply to the test points, and are defined as

20 $$ \begin{array}{*{20}c} {{\text{RMSE}}^{\left( k \right)} = \sqrt {\frac{1}{m}\mathop \sum \limits_{i = c + 1}^{c + m} \left( {y_{i}^{\left( k \right)} - \hat{y}_{i}^{\left( k \right)} } \right)^{2} } ,} \\ \end{array} $$

21 $$ \begin{array}{*{20}c} {{\text{MAPE}}^{\left( k \right)} = \sqrt {\frac{1}{m}\mathop \sum \limits_{i = c + 1}^{c + m} \left| {\frac{{y_{i}^{\left( k \right)} - \hat{y}_{i}^{\left( k \right)} }}{{y_{i}^{\left( k \right)} }}} \right|} ,} \\ \end{array} $$

where ${\mathrm{RMSE}}^{(k)}$ and ${\mathrm{MAPE}}^{(k)}$ are the RMSE and MAPE of the *k*-th SC, respectively. m is the number of points in test phase. Each test SC produces a RMSE and a MAPE. However, an accidental error in a single SC may result in a failure to accurately evaluate the model. Therefore, in order to further improve the evaluation ability of metrics, Average RMSE and Average MAPE are proposed, and defined as

22 $$ \begin{array}{*{20}c} {{\text{Average }}\;{\text{RMSE}} = \frac{1}{M}\mathop \sum \limits_{k = 1}^{M} {\text{RMSE}}^{\left( k \right)} ,} \\ \end{array} $$

23 $$ \begin{array}{*{20}c} {{\text{Average}}\;{\text{ MAPE}} = \frac{1}{M}\mathop \sum \limits_{k = 1}^{M} {\text{MAPE}}^{\left( k \right)} ,} \\ \end{array} $$

where M is the number of test SCs, which equals 22 in this study.

Since the output of GPR is a probability distribution, confidence intervals can be used to measure the model’s credibility. Confidence interval provides an interval estimation of the predicted value of a test point, and also represents the probability that the predicted value falls within a given interval. A confidence interval of 95% is usually adopted, and the bounds for Gaussian distribution are defined as^42^

24 $$ \begin{gathered} {\text{Upper bound}} = \mu + 1.96\sigma \hfill \\ {\text{Lower bound}} = \mu - 1.96\sigma , \hfill \\ \end{gathered} $$

where $\mu$ and $\sigma$ are the mean and standard deviation of the Gaussian distribution, respectively.

## Results and discussion

An SC labeled No. 10 is randomly selected from the test SCs, and the corresponding prediction result is analyzed. This randomly selected SC is representative since most of the SCs have a similar SOH trend. Cycle 500 is selected in most models as the boundary between training data and test data, implying that only the first 5% of the data (first 500 cycles) are used to predict the remaining 95% of the cycles. The data set is divided into three groups: preliminary data set, training data set, and test data set, where the preliminary data set comes from the previous SCs that have been tested before a new SC cycle. The training data set and test data set come from the 5% training cycles and the remaining 95% test cycles for a selected SC, respectively. In Table S1, seven models with different mean and covariance functions are used for comparison: (1) Model 1: with explicit logarithmic function, where the mean function is described as ${\text{m}}\left( x \right) = A{\text{log}}x + B$, and the covariance function is $\kappa_{SE} \left( {{\mathbf{x}},{\mathbf{x^{\prime}}}} \right)$; (2) Model 2: the mean function can be obtained from the preliminary data set, which is ${\text{m}}\left( x \right) = {\text{m}}_{pre} \left( x \right)$, and the covariance function is the same as Model 1; (3) Model 3: the mean function is the summation of Model 1 and Model 2, which is ${\text{m}}\left( x \right) = A{\text{log}}x + B + {\text{m}}_{pre} \left( x \right)$, and the covariance function is the same as Model 1; (4) Model 4: the mean function is the same as Model 1, but with different a covariance function $\kappa_{pre} + \kappa_{SE}$; (5) Model 5: the mean function is the same as Model 2, but with different a covariance function $\kappa_{pre} + \kappa_{SE}$; (6) Model 6: the mean function is the same as Model 3, but with a different covariance function $\kappa_{pre} + \kappa_{SE}$; and (7) Model 7: the mean function and covariance function are the same as Model 5, but use the first 1% of the cycles to predict the remaining 99% of the cycles.

**Figure 4:**
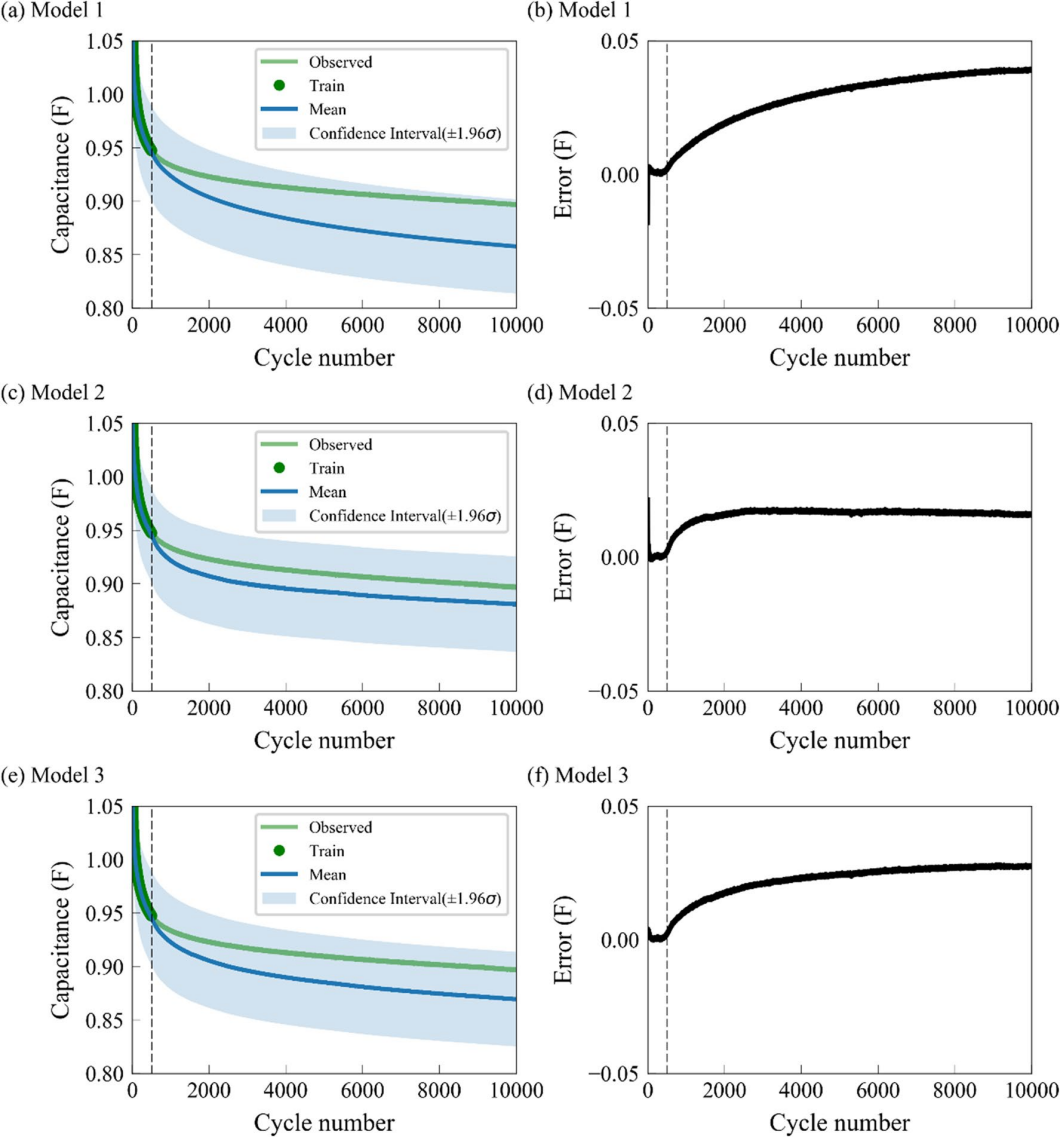
The capacitance predictions of three GPR models with different mean functions and covariance functions, where the first 5% cycles are used for training. (**a,c,e**) are the predicted capacitances of an SC for Models 1, 2, and 3, while (**b,d,f**) denote the predicted errors (observed − mean). The dotted line indicates the 500th cycle. The green and blue curves represent the observed and predicted mean capacitance, while the green dots, and blue shaded areas denote the predicted training data and confidence interval, respectively. The selection of ± 1.96 for confidence interval is based on Eq. (24).

In Table S1, the prediction result for Model 1 is the worst, where the Average RMSE is 0.021 F and the Average MAPE is 2.18%. The prediction result for Model 2 is the best, where the Average RMSE is 0.0139 F and the Average MAPE is 1.53%. The Average RMSE and Average MAPE for Model 3 is 0.0161 F and 1.72%, respectively. The results show that with the same covariance function, the mean function, consisting entirely of an implicit function, can achieve the lowest prediction error. Figure 4 shows the predicted capacitances and errors for models 1, 2, and 3, where the used covariance function of $\kappa_{SE} \left( {{\mathbf{x}},{\mathbf{x^{\prime}}}} \right)$ are the same and the mean function are different. The dotted line indicates the 500th cycle. Before the 500th cycle, the predicted results by GPR are in good agreement with our experiment. However, with an increase in the cycle numbers, the difference between GPR prediction and experiment increases, and the area of confidence interval increases. Figure 4 shows that the first 5% of the data can generate excellent prediction results for the first 500 cycles, but poor results for the remaining 95% of the cycles. As seen in Fig. 4b,d,f, the prediction errors increase with an increase in the cycle number, which is consistent with the characteristic of GPR model, that is, the farther it is away from the training point, the larger is the overall error. These results highlight the ability of the GPR-implicit function model to describe the degradation of SCs, especially for the first 5% of the data.

The optimized covariance functions in Models 1, 2, and 3 are based on the first 500 cycles. The degradation between the first 500 cycles and the remaining 9500 cycles is quite different; therefore, it is hard to capture the interaction between inputs and obtain a high SOH prediction accuracy. Rather than only considering the first 500 cycles in Models 1, 2, and 3, Models 4, 5, and 6 consider all cycles of an SC with prior knowledge from preliminary data set by introducing the implicit function $\kappa_{pre}$ in the covariance function. Among Models 4, 5, and 6, the prediction accuracy of Model 4 is the worst, with the predicted Average RMSE and Average MAPE being 0.0174 F and 1.77%, respectively. The prediction accuracy of Model 5 is the best, where the Average RMSE is 0.0056 F and the Average MAPE is 0.60%. The Average RMSE and Average MAPE of Model 6 is 0.0121 F and 1.26%, respectively. Compared with Models 1, 2, and 3, the prediction errors for Models 4, 5, and 6 decrease significantly after the introduction of implicit function. For example, by considering $\kappa_{pre}$ in the covariance function, Model 4 has a better performance and lower prediction error than Model 1, while Model 5 performs better than Model 2, and Model 6 performs better than Model 3, implying that the implicit function $\kappa_{pre}$ in the covariance function provides sufficient prior knowledge and strongly captures the interactions between inputs.

In addition to the GPR models discussed in this study, we also compare several non-gaussian data-driven methods as benchmark models, including two extrapolation models and three machine learning models (such as Auto Regression, Support Vector Machine and Random Forest models), please see Note 1 of the SM. Among these GPR and benchmark models, Model 5 is still the best performer, which further demonstrates the accuracy of implicit functions.

**Figure 5:**
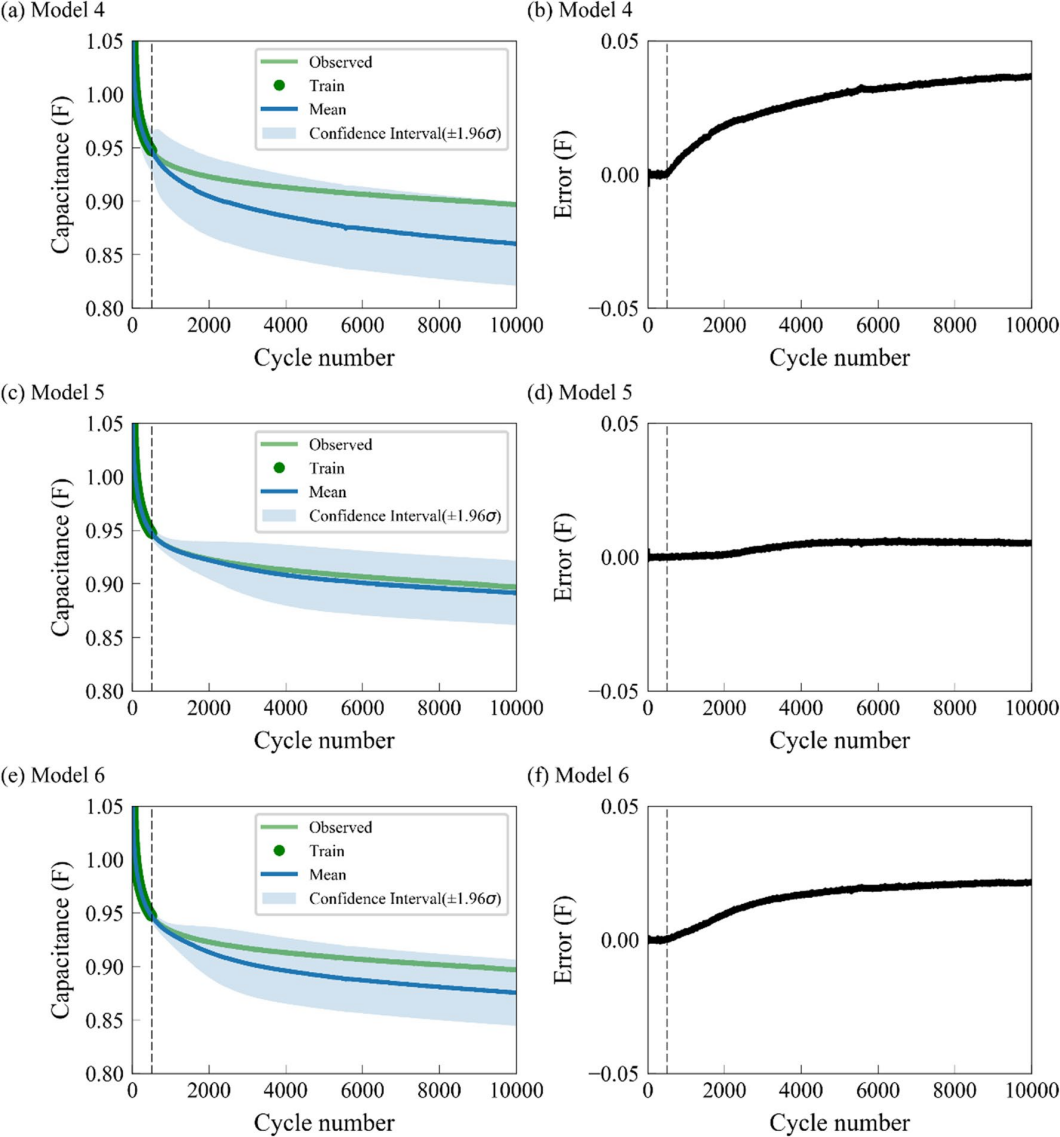
The predictions of the GPR models with different mean functions and compound covariance functions, where the first 5% cycles are used for training. The dotted line indicates the 500th cycle. (**a,c,e**) are the predicted capacitances of an SC for Models 4, 5, and 6, while (**b,d,f**) denote the predicted errors (observed − mean). The green and blue curves represent the observed and predicted mean capacitance, while the green dots and blue shaded areas denote the predicted training data and confidence interval, respectively. The selection of ± 1.96 for confidence interval is based on Eq. (24).

Figure 5 shows the predicted capacitances of an SC as functions of cycle numbers based on Models 4, 5, and 6. In Fig. 5a,c,e, the predicted mean capacitances (blue curves) based on Models 4, 5, and 6 are closer to the observed green curves, and the areas of confidence interval are smaller than the corresponding areas in Fig. 4. Figure 5 demonstrates that Models 4, 5, and 6, which consider all cycles of an SC with prior knowledge from the preliminary data set, are superior to Models 1, 2, and 3, which only consider the first 500 cycles. In Table S1, the best model is Model 5, with the lowest Average RMSE of 0.0056 and Average MAPE of 0.6%. This model uses the implicit function ${\text{m}}_{pre} \left( x \right)$ as a mean function and $\kappa_{pre} + \kappa_{SE}$ (implicit function + explicit function) as a covariance function. Models 8 and 9 are from the pioneering studies by Yang et al*.*^24^ and Liu et al*.*^22^, with predicted RMSEs of 0.0167 and 0.0332, respectively, having higher prediction errors than Model 5, further indicating that the implicit functions $\kappa_{pre} \left( {{\mathbf{x}},{\mathbf{x^{\prime}}}} \right)$ and ${\text{m}}_{pre} \left( x \right)$ can significantly enhance the SOH prediction accuracy. Furthermore, the above studies usually use at least 40% of the cycles as training data for SOH prediction, but Models 1, 2, 3, 4, 5, and 6 only use the first 5% (i.e. the first 500 cycles for training, the rest 9500 cycles for test) cycles as training data, exhibiting a strong and accurate long-term ability of GPR to predict the reliable SOH of energy storage devices and solve the data scarcity problem.

As a case study, Fig. 5 shows the strength of implicit mean and covariance functions for SOH prediction of a selected SC. To more reasonably investigate the model performances, we predict the SOH of all test SCs, and obtain their RMSEs and MAPEs (the values are shown in SM). The predicted Average RMSE and Average MAPE of all SCs are 0.0056F and 0.60%, respectively.

**Figure 6:**
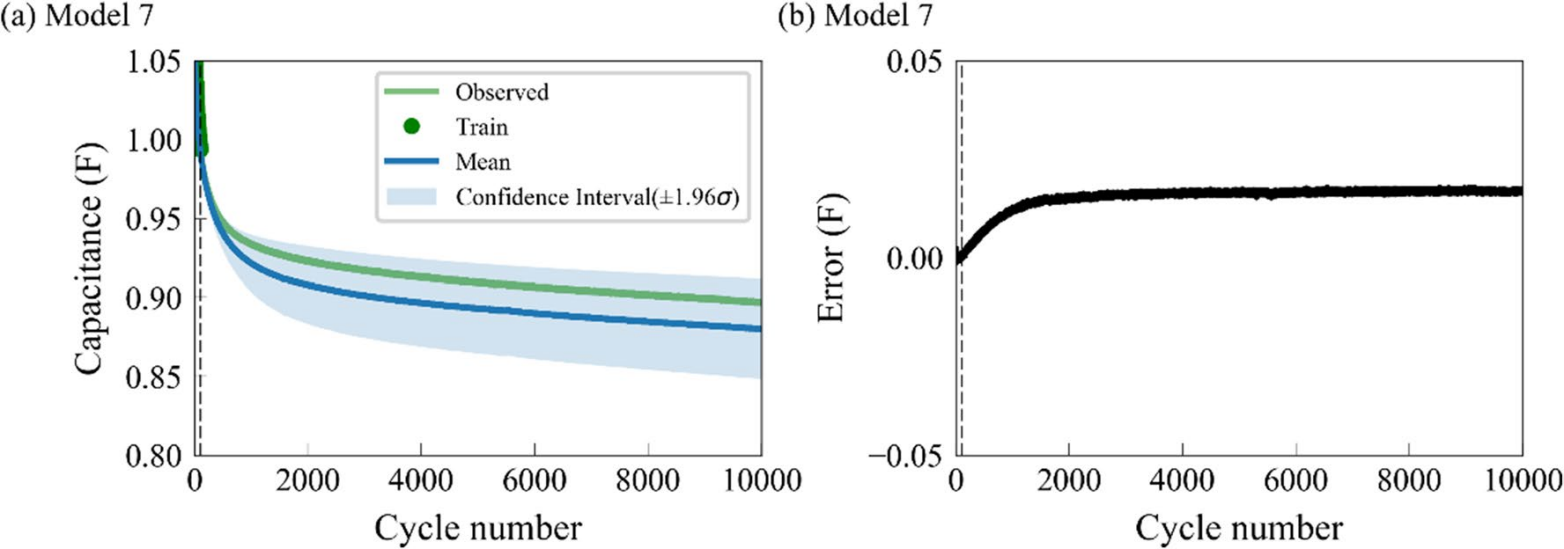
The prediction results of the Model 7, where only 1% of the cycles are used for training. The dotted line indicates the 100th cycle. (**a**) The predicted capacitance for Model 7 as a function of cycle number. (**b**) The predicted error (observed − mean) for Model 7. The curves and blue shaded areas denote the predicted training data and confidence interval, respectively.

In Table S1, Model 5 has the best performance with the first 5% cycles for training, which demonstrates the strength of the implicit function for SOH prediction. To further test the capacity of implicit function, Model 7 shows the SOH prediction using only the first 1% of the cycles as training data, with the same mean function and covariance function as Model 5. As shown in Fig. 6, the dotted lines indicate the 100th cycle, the first 1% of total SC cycles. For Model 7, the predicted RMSE is 0.0156 F and the MAPE is 1.69%, and the Average RMSE and Average MAPE of all SCs are 0.0094 F and 1.01%, respectively (Table S1). Although less information is provided, the GRP-implicit function model can still capture the SOH trend, and the performance is better than all models excluding Model 5 that uses the first 500 cycles. Compared with Model 5, the prediction error of Model 7 is slightly larger, but the number of training data cycles is only 1%, indicating that it does not require a large number of training data, and can ease the problem of data scarcity in SOH prediction.

In addition, models (model 10, model 11, model 12, model 13, model 14 and model 15) with different kernel functions except SE function ($\kappa_{SE} \left( {{\mathbf{x}},{\mathbf{x^{\prime}}}} \right)$) are also applied as benchmark models to illustrate the universality of the method reported in this paper. Two kernel functions are chosen, including  function and rational quadratic function (RQ function), which are both common kernel functions in the prediction of SOH^43^. The formula of  function is shown as follow:25where γ is a hyperparameter to reflect the smoothness and ${\mathcal{R}}_{\gamma }$ represents the Bessel function. The  was a widely used for  functions as kernel function of GPR^43^. Therefore, the value of $\gamma$ was set as 2.5.

Along with SE function and  function, the RQ function is also a popular kernel function for the GPR. The formula of RQ function can be obtained as follow:

26 $$ \begin{array}{*{20}c} {\kappa_{RQ} \left( {{\mathbf{x}},{\mathbf{x^{\prime}}}} \right) = \delta_{RQ}^{2} \left( {1 + \frac{{\left( {k - k^{^{\prime}} } \right)^{2} }}{{2\alpha l_{RQ}^{2} }}} \right)^{ - \alpha } ,} \\ \end{array} $$

where α reflects the relative weight of scale changes. $\delta_{RQ}$ and $l_{RQ}$ are hyper-parameters for controlling the vertical scale and length scale of the Gaussian function, respectively.

The kernel functions of model 10, model 11 and model 12 include  function ($\kappa_{MA} \left( {{\mathbf{x}},{\mathbf{x^{\prime}}}} \right)$). The kernel functions of model 13, model 14 and model 15 include rational quadratic function (RQ function, $\kappa_{RQ} \left( {{\mathbf{x}},{\mathbf{x^{\prime}}}} \right)$). The average RMSE and average MAPE of these six models are shown in Table S1. Through the comparison between errors of these models, the superiority of GPR with implicit functions can be further demonstrated: (1) The mean function of model 10 and model 11 is the same. The kernel function of model 11 is $\kappa_{pre} \left( {{\mathbf{x}},{\mathbf{x^{\prime}}}} \right) + \kappa_{MA} \left( {{\mathbf{x}},{\mathbf{x^{\prime}}}} \right)$ (include the implicit function) and the kernel function of model 10 is $\kappa_{MA} \left( {{\mathbf{x}},{\mathbf{x^{\prime}}}} \right)$ (does not include the implicit function). As shown in Table S1, the average RMSE and average MAPE of model 11 (0.0323F, 3.17%) is much lower than model 10 (0.0715F, 7.11%), demonstrating the higher performance of GPR with implicit function as kernel function. (2)The kernel function of model 12 and model 11 is the same. The mean function of model 11 is ${\text{m}}_{pre} \left( x \right)$ (an implicit function) and the mean function of model 10 is $A{\text{log}}x + B$(an explicit function). Similarly, the average RMSE and average MAPE of model 11 is much lower than that of model 12 (0.1148F, 11.19%), demonstrating the higher performance of GPR as a mean function.

The comparison between models 10, 11 and 12 discussed above illustrated that the GPRs with  function in the kernel functions conform to the conclusion of these paper. Similar comparation can be performed between models 13, 14 and 15, which are based on the kernel functions with RQ function. The better performance of model 14 (average RMSE = 0.0150F, average MAPE = 1.59%) than model 13 (average RMSE = 0.0201F, average MAPE = 2.12%) and model 15 (average RMSE = 0.221F, average MAPE = 2.27%) demonstrated that the modified functions has good applicability to the RQ function based GPR method.

## Conclusion

Accurate SOH prediction can grasp the working state of SCs and avoid failure in advance. This work proposes an implicit function learning method to predict the SOH of SCs based on Gaussian process regression, where a preliminary data set is considered in the mean and covariance functions, and only 5% of the cycles are used as training data to predict the remaining 95% of the cycles. The predicted errors of the GPR-implicit function method have an Average RMSE of 0.0056 F and an Average MAPE of 0.06%, which are much lower than traditional SOH predictions with only explicit functions. The present work demonstrates the GPR-implicit functions can effectively compensate or replace the GPR-explicit functions for SOH prediction, with advantages of high precision and low data requirement. We further investigate SOH prediction using fewer cycles (1% of the cycles) as the training data, and produce reasonable prediction errors, indicating that the GPR-implicit function learning has a strong ability to predict SOH.

In the future, some additional extensions can be explored to improve the prediction performance of the GPR-implicit function method, including (1) using more comprehensive feature descriptors such as voltage, current, and temperature, or their mathematical transformations; (2) using different explicit functions, for example, an exponential function can be used as a mean function, and the Matérn function can be used as a covariance function; (3) optimizing the coefficients of explicit and implicit functions. (4) The proposed method follows a sequence from simple to complex. Since the attenuation process of supercapacitors is relatively simple, the proposed method is applied to supercapacitors first. In the future, we will update and optimize the algorithm to adapt to the attenuation process of more complex electrical systems, including a variety of different batteries. We hope that this work can promote the study of SOH prediction for supercapacitors and other energy storage devices.

## Supplementary Information

Supplementary Information.

## Data Availability

The raw data required to reproduce these findings are available to download from https://dx.doi.org/10.6084/m9.figshare.11522082.
