# Supplementary material for: High precision implicit function learning for forecasting supercapacitor state of health based on Gaussian process regression
Source: Sci Rep. 2021 Jun 8;11:12112. doi: 10.1038/s41598-021-91241-z (PMC8187390; doi:10.1038/s41598-021-91241-z)
Supplement: Supplementary file 1 — Supplementary Information. [file 41598_2021_91241_MOESM1_ESM.pdf]

Supplementary Materials (SM) for High Precision Implicit Function Learning for Forecasting Supercapacitor State of Health Based on Gaussian Process Regression

Jiahao Ren[1,2], Junfei Cai [1,2] and Jinjin Li[1,2]*

[1]National Key Laboratory of Science and Technology on Micro/Nano Fabrication, Shanghai Jiao Tong University, Shanghai, 200240, China

[2]Department of Micro/Nano Electronics, School of Electronic Information and Electrical Engineering, Shanghai Jiao Tong University, Shanghai, 200240, China

*Correspondence to: Jinjin Li (lijinjin@sjtu.edu.cn)

Table S1 Comparison of different GRP models performed in this study with different mean and covariance functions. $\mathrm{m}_{pre}(x)$ and $\kappa_{pre}(\mathbf{x},\mathbf{x}')$ are implicit functions, while the rest are explicit functions. $\kappa_{SE}(\mathbf{x},\mathbf{x}')$ is the squared exponential covariance function. $\kappa_{MA}(\mathbf{x},\mathbf{x}')$ is the $Matérn$ covariance function. $\kappa_{RQ}(\mathbf{x},\mathbf{x}')$ is the rational quadratic covariance function.

Model label	Mean function $\mathrm{m}(x)$	[a]Covariance function $\kappa(\mathbf{x},\mathbf{x}')$	[b]RMSE	[b]MAPE (%)	[c]Average (Median) RMSE	[c]Average (Median) MAPE (%)
1	$A\log x + B$	$\kappa_{SE}(\mathbf{x},\mathbf{x}')$	0.0308	3.24	0.0210 (0.0210)	2.18 (2.24)
2	$\mathrm{m}_{pre}(x)$	$\kappa_{SE}(\mathbf{x},\mathbf{x}')$	0.0162	1.76	0.0139 (0.0153)	1.53 (1.67)
3	$A\log x + B$ $+ \mathrm{m}_{pre}(x)$	$\kappa_{SE}(\mathbf{x},\mathbf{x}')$	0.0233	2.49	0.0161 (0.0191)	1.72 (2.05)
4	$A\log x + B$	$\kappa_{pre}(\mathbf{x},\mathbf{x}')$ $+ \kappa_{SE}(\mathbf{x},\mathbf{x}')$	0.0288	3.02	0.0174 (0.0188)	1.77 (1.88)
5	$\mathrm{m}_{pre}(x)$	$\kappa_{pre}(\mathbf{x},\mathbf{x}')$ $+ \kappa_{SE}(\mathbf{x},\mathbf{x}')$	0.0046	0.46	0.0056 (0.0054)	0.60 (0.57)
6	$A\log x + B$ $+ \mathrm{m}_{pre}(x)$	$\kappa_{pre}(\mathbf{x},\mathbf{x}')$ $+ \kappa_{SE}(\mathbf{x},\mathbf{x}')$	0.0172	1.78	0.0121 (0.0133)	1.26 (1.37)
[d]7	$\mathrm{m}_{pre}(x)$	$\kappa_{pre}(\mathbf{x},\mathbf{x}')$ $+ \kappa_{SE}(\mathbf{x},\mathbf{x}')$	0.0157	1.69	0.0094 (0.0067)	1.01 (0.71)
[e]8	0	k_f	/	/	0.0167 (0.0122)	/

[f]9	*Ax+B*	k_f’	/	/	0.0332 (0.0173)	4.50 (1.70)
10	$\mathrm{m}_{pre}(x)$	$\kappa_{MA}(\mathbf{x},\mathbf{x}')$	0.0274	2.79	0.0715 (0.0566)	7.11 (5.42)
11	$\mathrm{m}_{pre}(x)$	$\kappa_{pre}(\mathbf{x},\mathbf{x}')$ $+\kappa_{MA}(\mathbf{x},\mathbf{x}')$	0.0063	5.40	0.0323 (0.0211)	3.17 (2.04)
12	$A\log x+B$	$\kappa_{pre}(\mathbf{x},\mathbf{x}')$ $+\kappa_{MA}(\mathbf{x},\mathbf{x}')$	0.1094	9.88	0.1148 (0.1167)	11.19 (11.29)
13	$\mathrm{m}_{pre}(x)$	$\kappa_{RQ}(\mathbf{x},\mathbf{x}')$	0.0251	2.48	0.0201 (0.0153)	2.12 (1.62)
14	$\mathrm{m}_{pre}(x)$	$\kappa_{pre}(\mathbf{x},\mathbf{x}')$ $+\kappa_{RQ}(\mathbf{x},\mathbf{x}')$	0.0161	1.75	0.0150 (0.0155)	1.59 (1.65)
15	$A\log x+B$	$\kappa_{pre}(\mathbf{x},\mathbf{x}')$ $+\kappa_{RQ}(\mathbf{x},\mathbf{x}')$	0.0310	0.0327	0.0221 (0.0220)	2.27 (2.26)

[a] The noise term is ignored for brevity

[b] Refer to the error of SC No. 10

[c] Refer to the average error of all test SCs. The value in brackets is the median.

[d] The first 100 cycles are used for prediction

[e] Provided by ref[24]

[f] Provided by ref[22]

$k_f = \sigma_{f_1}^2 \exp\left(-\frac{(x_i-x_j)^2}{2l_1^2}\right) + \sigma_{f_2}^2 \exp\left(-\frac{(x_i-x_j)^2}{2l_2^2}\right) + \sigma_n^2 \delta(x_i, x_j)$

$k_f' = \sigma_{f_1}^2 \exp\left(-\frac{(x_i-x_j)^2}{2l_1^2}\right) + \sigma_{f_2}^2 \exp\left(-\frac{2}{l_2^2} sin^2\left(\frac{\omega}{2\pi}(x_i - x_j)\right)\right)$

Table S2. The RMSE and MAPE of all test SCs of model 1. The first 500 cycles are used for training while the subsequent 9,500 cycles are used for prediction. The No.10 SC is selected for illustration in the main paper.

Number	RMSE(F)	MAPE(%)
1	0.030906	3.2544
2	0.006165	0.6286
3	0.029915	3.1741
4	0.009140	0.9239
5	0.009124	0.8801
6	0.022717	2.3848
7	0.008233	0.7841
8	0.010495	0.9819
9	0.030727	3.2564
10	0.030808	3.2371
11	0.015400	1.5247
12	0.019313	2.0951
13	0.032355	3.4081
14	0.033627	3.5228
15	0.013884	1.3458
16	0.032743	3.3867
17	0.017318	1.7091
18	0.008323	0.7347
19	0.029733	3.1301
20	0.017313	1.8699
21	0.030246	3.1952
22	0.023557	2.4580
Mean	0.021002	2.1766
Median	0.021015	2.2399

Table S3. The RMSE and MAPE of all test SCs of model 2. The first 500 cycles are used for training while the subsequent 9,500 cycles are used for prediction. The No.10 SC is selected for illustration in the main paper.

Number	RMSE(F)	MAPE(%)
1	0.016270	1.7777
2	0.012382	1.3639
3	0.015472	1.6943
4	0.009715	1.0915
5	0.007633	0.8343
6	0.008143	0.8942
7	0.008044	0.8702
8	0.013422	1.4785
9	0.016323	1.7777
10	0.016161	1.7592
11	0.002252	0.1726
12	0.031754	3.6592
13	0.017737	1.9334
14	0.018884	2.0504
15	0.002333	0.2468
16	0.017759	1.9155
17	0.001838	0.1877
18	0.019240	2.1636
19	0.015073	1.6503
20	0.029910	3.4305
21	0.015672	1.7159
22	0.008774	0.9672
Mean	0.021002	1.528844
Median	0.021015	1.672289

Table S4. The RMSE and MAPE of all test SCs of model 3. The first 500 cycles are used for training while the subsequent 9,500 cycles are used for prediction. The No.10 SC is selected for illustration in the main paper.

Number	RMSE(F)	MAPE(%)
1	0.023443	2.5162
2	0.005938	0.5993
3	0.022512	2.4343
4	0.005348	0.4287
5	0.003219	0.3055
6	0.015191	1.6393
7	0.002420	0.2191
8	0.009209	0.7257
9	0.023343	2.5171
10	0.023341	2.4984
11	0.007793	0.7801
12	0.025106	2.8774
13	0.024907	2.6709
14	0.026140	2.7867
15	0.006222	0.6040
16	0.025166	2.6511
17	0.009553	0.9452
18	0.012622	1.3935
19	0.022255	2.3903
20	0.023170	2.6506
21	0.022800	2.4557
22	0.015987	1.7127
Mean	0.016167	1.7183
Median	0.019121	2.0515

Table S5. The RMSE and MAPE of all test SCs of model 4. The first 500 cycles are used for training while the subsequent 9,500 cycles are used for prediction. The No.10 SC is selected for illustration in the main paper.

Number	RMSE(F)	MAPE(%)
1	0.025957	2.7090
2	0.006450	0.6490
3	0.018380	1.8505
4	0.020111	2.1114
5	0.010548	1.0118
6	0.010658	1.0390
7	0.007021	0.6836
8	0.027679	2.9670
9	0.025151	2.6199
10	0.028825	3.0195
11	0.020066	2.0451
12	0.006384	0.5795
13	0.023593	2.4282
14	0.026009	2.6660
15	0.012403	1.2200
16	0.026172	2.6916
17	0.016570	1.6651
18	0.006845	0.6050
19	0.020359	2.0506
20	0.009724	0.9521
21	0.019177	1.9083
22	0.015321	1.5314
Mean	0.017427	1.7729
Median	0.018779	1.8794

Table S6. The RMSE and MAPE of all test SCs of model 5. The first 500 cycles are used for training while the subsequent 9,500 cycles are used for prediction. The No.10 SC is selected for illustration in the main paper.

Number	RMSE (F)	MAPE (%)
1	0.004344	0.4442
2	0.005713	0.6251
3	0.007671	0.8151
4	0.008977	0.9606
5	0.000693	0.0611
6	0.000883	0.0816
7	0.004607	0.4966
8	0.008729	0.9234
9	0.006613	0.7137
10	0.004596	0.4568
11	0.001081	0.1104
12	0.009536	1.0771
13	0.004208	0.4400
14	0.005101	0.5209
15	0.003503	0.3546
16	0.002822	0.2644
17	0.007565	0.8101
18	0.008221	0.9228
19	0.001413	0.1302
20	0.012159	1.3592
21	0.006600	0.6633
22	0.008337	0.8973
Mean	0.005608	0.5968
Median	0.005407	0.5730

Table S7. The RMSE and MAPE of all test SCs of model 6. The first 500 cycles are used for training while the subsequent 9,500 cycles are used for prediction. The No.10 SC is selected for illustration in the main paper.

Number	RMSE(F)	MAPE(%)
1	0.014138	1.4708
2	0.007054	0.7352
3	0.020794	2.1829
4	0.002946	0.2855
5	0.012290	1.3184
6	0.008804	0.9340
7	0.008074	0.8567
8	0.007252	0.6208
9	0.018935	2.0054
10	0.013234	1.3819
11	0.013603	1.4345
12	0.003013	0.2993
13	0.016818	1.7515
14	0.017718	1.8306
15	0.016072	1.6975
16	0.015343	1.5699
17	0.020036	2.1380
18	0.002736	0.2537
19	0.013364	1.3575
20	0.004356	0.4577
21	0.018400	1.9018
22	0.011537	1.1762
Mean	0.012114	1.2573
Median	0.013299	1.3697

Table S8. The RMSE and MAPE of all test SCs of model 7. The first 100 cycles are used for training while the subsequent 9,900 cycles are used for prediction. The No.10 SC is selected for illustration in the main paper.

Number	RMSE (F)	MAPE (%)
1	0.015850	1.7050
2	0.006893	0.7460
3	0.001134	0.1013
4	0.017903	1.9681
5	0.001275	0.1217
6	0.002907	0.2528
7	0.005563	0.5978
8	0.025007	2.7800
9	0.005982	0.6314
10	0.015683	1.6911
11	0.007567	0.8240
12	0.013585	1.5199
13	0.006470	0.6818
14	0.010953	1.1487
15	0.005296	0.5729
16	0.008375	0.8789
17	0.001794	0.1758
18	0.018865	2.0959
19	0.004840	0.4969
20	0.021631	2.4347
21	0.002762	0.2531
22	0.005368	0.5598
Mean	0.009350	1.0108
Median	0.006682	0.7139

Table S9. The average(median) RMSE and MAPE of all test SCs of benchmarking models mentioned in Note 1.

Model name	Average (Median) RMSE	Average (Median) MAPE (%)
Power	0.047743<br>(0.044158)	4.8175<br>(4.4327)
Logarithmic	0.018299<br>(0.018573)	1.8402<br>(1.8035)
AR	0.009238<br>(0.0097195)	1.0143<br>(1.0533)
SVM	0.011317<br>(0.010057)	1.2547<br>(1.0909)
RF	0.007052<br>(0.005999)	0.7526<br>(0.6347)

Table S10. The RMSE and MAPE of all test SCs of model 10. The first 500 cycles are used for training while the subsequent 9500 cycles are used for prediction.

Number	RMSE (F)	MAPE (%)
1	0.0123	1.15%
2	0.1900	18.67%
3	0.0175	1.85%
4	0.0055	0.51%
5	0.0103	1.05%
6	0.0632	6.32%
7	0.0087	0.87%
8	0.1029	10.42%
9	0.0163	1.74%
10	0.0274	2.79%
11	0.0720	7.23%
12	0.0086	0.83%
13	0.0657	17.36%
14	0.1796	12.77%
15	0.1305	1.79%
16	0.0171	15.83%
17	0.1582	13.18%
18	0.1280	16.60%
19	0.1667	13.78%
20	0.1348	0.84%
21	0.0090	4.51%
22	0.0500	6.41%
Mean	0.0715	7.11
Median	0.0566	5.42

Table S11. The RMSE and MAPE of all test SCs of model 11. The first 500 cycles are used for training while the subsequent 9500 cycles are used for prediction. The No.10 SC is selected for illustration in the main paper.

Number	RMSE (F)	MAPE (%)
1	0.0032	0.32%
2	0.0723	7.22%
3	0.0164	1.72%
4	0.0121	1.20%
5	0.0164	1.70%
6	0.0369	3.69%
7	0.0351	3.79%
8	0.1877	18.47%
9	0.0116	0.97%
10	0.0063	0.54%
11	0.0180	1.67%
12	0.0107	1.18%
13	0.0443	4.30%
14	0.0213	2.08%
15	0.0209	2.17%
16	0.0222	2.01%
17	0.0303	2.78%
18	0.0397	4.00%
19	0.0093	0.97%
20	0.0318	3.28%
21	0.0154	1.40%
22	0.0494	4.51%
Mean	0.0323	3.17
Median	0.0211	2.04

Table S12. The RMSE and MAPE of all test SCs of model 12. The first 500 cycles are used for training while the subsequent 9500 cycles are used for prediction.

Number	RMSE (F)	MAPE (%)
1	0.1094	10.46%
2	0.1109	10.86%
3	0.0432	3.92%
4	0.1297	12.78%
5	0.1260	12.24%
6	0.0982	9.18%
7	0.1552	15.42%
8	0.1466	14.86%
9	0.1920	19.43%
10	0.1032	9.88%
11	0.0960	9.30%
12	0.1243	12.53%
13	0.0697	6.47%
14	0.1351	12.78%
15	0.0936	8.82%
16	0.0919	8.71%
17	0.1447	14.24%
18	0.0745	7.21%
19	0.1226	11.72%
20	0.0825	8.08%
21	0.1328	12.77%
22	0.1456	14.48%
Mean	0.1148	11.19
Median	0.1167	11.29

Table S13. The RMSE and MAPE of all test SCs of model 13. The first 500 cycles are used for training while the subsequent 9500 cycles are used for prediction.

Number	RMSE (F)	MAPE (%)
1	0.0663	6.21%
2	0.0225	2.47%
3	0.0030	0.32%
4	0.0023	0.25%
5	0.0175	1.91%
6	0.0804	8.72%
7	0.0163	1.74%
8	0.0068	0.76%
9	0.0107	1.10%
10	0.0251	2.48%
11	0.0023	0.22%
12	0.0220	2.44%
13	0.0085	0.89%
14	0.0047	0.49%
15	0.0170	1.82%
16	0.0386	4.10%
17	0.0144	1.51%
18	0.0246	2.70%
19	0.0055	0.59%
20	0.0321	3.56%
21	0.0141	1.44%
22	0.0088	0.95%
Mean	0.0201	2.12
Median	0.0153	1.62

Table S14. The RMSE and MAPE of all test SCs of model 14. The first 500 cycles are used for training while the subsequent 9500 cycles are used for prediction.

Number	RMSE (F)	MAPE (%)
1	0.0129	1.41%
2	0.0174	1.86%
3	0.0143	1.55%
4	0.0150	1.47%
5	0.0144	1.47%
6	0.0106	0.66%
7	0.0115	1.28%
8	0.0120	1.34%
9	0.0167	1.81%
10	0.0161	1.75%
11	0.0044	0.24%
12	0.0310	3.56%
13	0.0185	2.01%
14	0.0192	2.09%
15	0.0043	0.26%
16	0.0192	2.08%
17	0.0016	0.15%
18	0.0191	2.15%
19	0.0170	1.82%
20	0.0302	3.47%
21	0.0161	1.76%
22	0.0087	0.95%
Mean	0.0150	1.59
Median	0.0155	1.65

Table S15. The RMSE and MAPE of all test SCs of model 15. The first 500 cycles are used for training while the subsequent 9500 cycles are used for prediction.

Number	RMSE (F)	MAPE (%)
1	0.0279	2.87%
2	0.0086	0.70%
3	0.0294	3.08%
4	0.0285	3.06%
5	0.0110	1.05%
6	0.0206	2.10%
7	0.0075	0.74%
8	0.0106	1.00%
9	0.0314	3.34%
10	0.0310	3.27%
11	0.0169	1.66%
12	0.0193	2.05%
13	0.0334	3.53%
14	0.0338	3.51%
15	0.0115	1.14%
16	0.0341	3.52%
17	0.0175	1.79%
18	0.0092	0.76%
19	0.0308	3.24%
20	0.0191	1.96%
21	0.0308	3.23%
22	0.0234	2.42%
Mean	0.0221	2.27
Median	0.0220	2.26

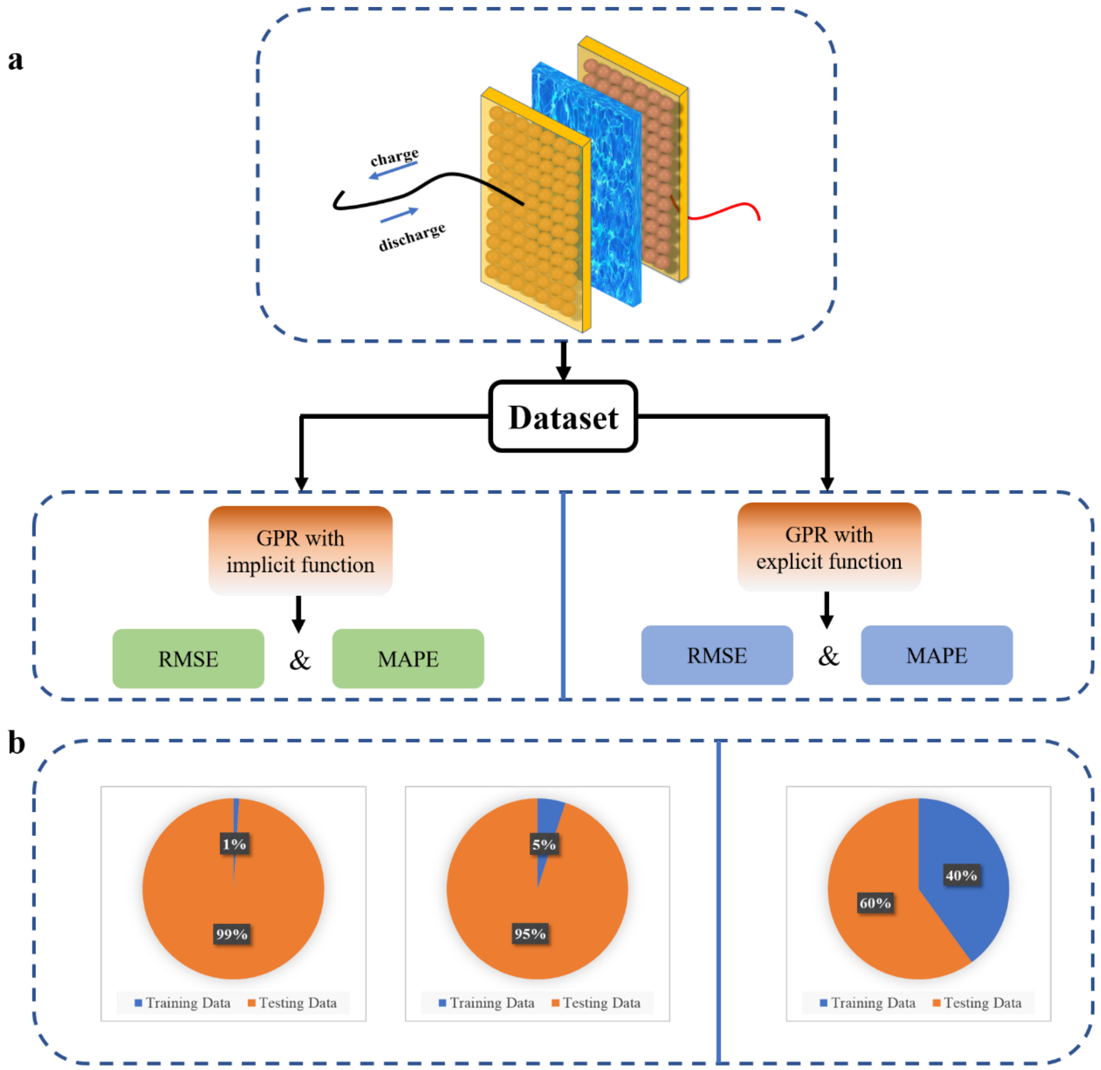

Fig. S1 Research framework. **a** Comparison of GPR with implicit and explicit functions. **b** Comparison of accuracy when using different proportions of data as the training set.

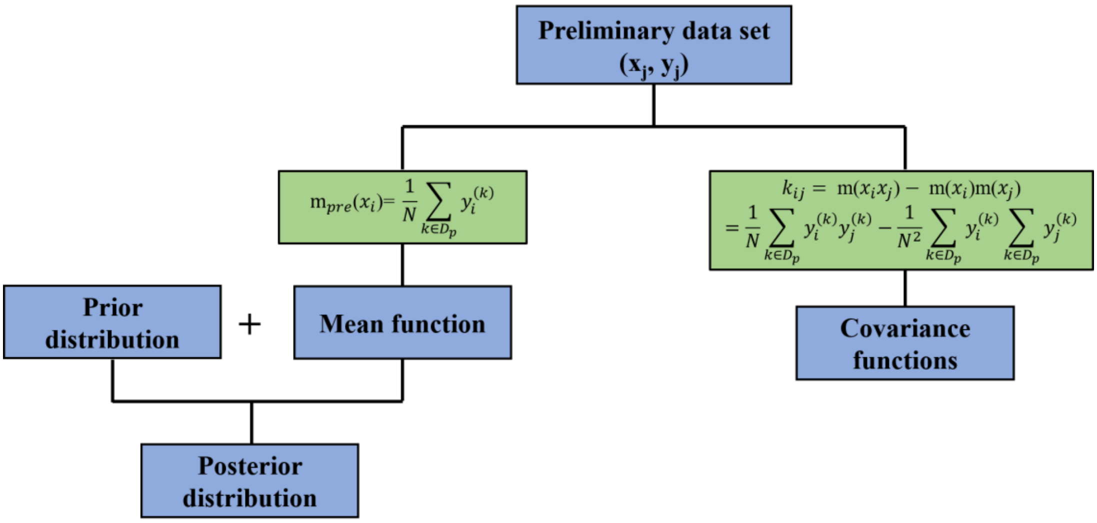

Fig. S2 Flow chart of obtaining the mean and covariance functions from the preliminary data set.

Note 1. Benchmarking models

Additional two function extrapolation models and three machine learning models are performed to compare the GPR models developed in this study.

1. "Power": The decay curve of the first 500 cycles is fitted by the power function of $y = aN^b + c$ where the a, b and c are parameters, N is number of cycle, and y is capacitance. Then the SOH of rest 9,500 cycles are calculated by the power function.

2. "Logarithmic": The decay curve of the first 500 cycles is fitted by the logarithmic function of $y = a\log N + b$ where the a and b are parameters, N is number of cycle, and y is capacitance. Then the SOH of rest 9,500 cycles are calculated by the logarithmic function.

3. "AR": AutoRegression method with the input of capacitance of the first 500 cycles reduced by principal component analysis. The SOH is a linear combination of input features.

4. "SVM": Support vector machine method with the input of capacitance of the first 500 cycles reduced by principal component analysis.

5. "RF": Random Forest method with the input of capacitance of the first 500 cycles reduced by principal component analysis.

The "Power" and "Logarithmic" models use function to fit the decay curve of the first 500 cycles and then predict the SOH on the whole life, where the training set is not required. The three machine learning models, "AR", "SVM" and "RF" use the capacitance of the first 500 cycles reduced by principal component analysis(PCA) as multidimensional input features. The input features firstly is up to 500 dimensions but such a high dimensional features will lead to optimization failure. Therefore, the PCA is performed and the input features is reduced from 500 to 16 dimensions, while

most of the information is retained. All additional models are performed with the same metrics as GPR models in the main text, whose results are shown in Table S8.

The errors of “Power” are 0.0477 average RMSE and 4.82% average MAPE, and the errors of “Logarithmic” are 0.0183 average RMSE and 1.84% average MAPE, and the errors of “AR” are 0.0092 average RMSE and 1.01% average MAPE, and the errors of “SVM” are 0.0113 average RMSE and 1.25% average MAPE, and the errors of “RF” are 0.0072 average RMSE and 0.76% average MAPE. The two function extrapolation models are too simple and do not use the information of the training set, and naturally perform poorly. The three machine learning models capture the internal relationship between features and output from training set and outperform all GPR models using the first 500 cycles as prior knowledge except for Model 5 , and the average MAPE of Model 5 is about 0.15% smaller than the best “RF” model. By improving the GPR method, we have successfully improved the prediction accuracy of GPR by implicit functions which surpasses some other machine learning models. More importantly, we expand the source of the mean functions and covariance functions when applying GPR method and prove its high accuracy. This contribution will provide reference for other studies in the future.
